# Histopathological Evaluation of Contrast-Induced Acute Kidney Injury Rodent Models

**DOI:** 10.1155/2016/3763250

**Published:** 2016-11-16

**Authors:** Norbert Kiss, Péter Hamar

**Affiliations:** Institute of Pathophysiology, Semmelweis University, Budapest, Hungary

## Abstract

Contrast-induced acute kidney injury (CI-AKI) can occur in 3–25% of patients receiving radiocontrast material (RCM) despite appropriate preventive measures. Often patients with an atherosclerotic vasculature have to receive large doses of RCM. Thus, animal studies to uncover the exact pathomechanism of CI-AKI are needed. Sensitive and specific histologic end-points are lacking; thus in the present review we summarize the histologic appearance of different rodent models of CI-AKI. Single injection of RCM causes overt renal damage only in rabbits. Rats and mice need an additional insult to the kidney to establish a clinically manifest CI-AKI. In this review we demonstrate that the concentrating ability of the kidney may be responsible for species differences in sensitivity to CI-AKI. The most commonly held theory about the pathomechanism of CI-AKI is tubular cell injury due to medullary hypoxia. Thus, the most common additional insult in rats and mice is some kind of ischemia. The histologic appearance is tubular epithelial cell (TEC) damage; however severe TEC damage is only seen if RCM is combined by additional ischemia. TEC vacuolization is the first sign of CI-AKI, as it is a consequence of RCM pinocytosis and lysosomal fusion; however it is not sensitive as it does not correlate with renal function and is not specific as other forms of TEC damage also cause vacuolization. In conclusion, histopathology alone is insufficient and functional parameters and molecular biomarkers are needed to closely monitor CI-AKI in rodent experiments.

## 1. Introduction

**Figure 1:**
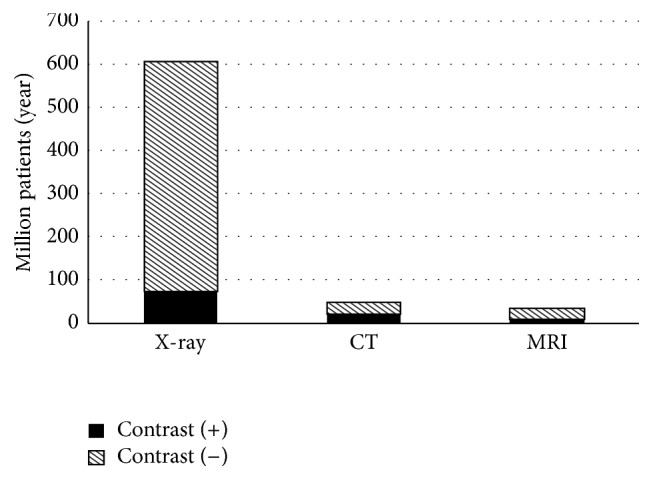
Use of contrast materials in the USA. The most common cause of intravenous iodinated contrast media [27] is X-ray (75 million/year) while about 50% of the CT and MRI studies are also contrast enhanced accounting for another 38 million patients/year. The degree of contrast utilization is expected to increase in the future [28]. The global market for radiopharmaceuticals is $4.5 billion in 2015 and is projected to reach US$ 6.63 billion by 2017 [29]. The iodinated, injectable contrast agents segment is expected to account for the largest share of the contrast agents market [30].

Contrast-induced acute kidney injury (CI-AKI) is defined as an increase of >25% or >0.5 mg/dL (44 *μ*mol/L) serum creatinine (*S*_Cr_) from baseline within 48−72 hours following intravenous injection of iodine-based radiocontrast material (RCM) if other causes of renal impairment can be excluded [1–3]. CI-AKI is the third most common cause of hospital acquired acute renal failure [2, 4, 5] and is responsible for about 10–12% of the cases [2, 6–8]. Renal function may deteriorate after RCM use even in the absence of overt CI-AKI [9, 10]. CI-AKI was first reported in 1942 [11]. The rise in the use of intravenous RCM for computer tomography (CT) and magnetic resonance imaging (MRI) and angiography [5] (Figure 1) led to the recognition of CI-AKI as the most important complication of iodinated RCM administration. Although contrast enhanced X-ray is the most common indication of RCM use (Figure 1), percutaneous coronary angiography (in the USA: 1.4–1.3 million/year between 1997 and 2010 [12]) is the primary cause of CI-AKI [4, 5]. Although hydration is considered to be the most effective preventive measure [13, 14], CI-AKI may develop in 3–25% of patients despite appropriate hydration [15] and further preventive measures such as antioxidants [2, 16] or statins [4, 17]. However, the affected patient population is much larger considering subclinical cases [9]. Furthermore, RCM administration in patients with high risk of CI-AKI such as preexisting renal problems [18] or the use of large RCM doses [19] is often unavoidable, which underscores the need for effective prevention strategies.

Although hypoxia of the renal medulla [20–22] due to reduction of renal blood flow (RBF) especially in peritubular capillaries [23] and consequent oxidative stress are thought to be the major effectors of CI-AKI [13, 24–26], the exact* pathomechanism* is unknown [15, 20]. Thus CI-AKI is a subject of intense research. In-depth analysis of molecular pathophysiology requires animal models. Therefore, different rodent models, such as mice, rats, and also rabbits, are commonly applied in CI-AKI research. Histopathological analysis of the kidney can provide a definitive end-point in various experimental settings. However, the pathological appearance can vary between experimental models and a detailed description of CI-AKI histopathology is not available. Many of the experimental models also involve additional insults beside RCM; hence it is relevant to differentiate between specific and nonspecific histopathological lesions. In the present review we summarize the histopathological findings in CI-AKI rodent models.

## 2. RCM Classes and Their Osmolality and Viscosity

The* type* [5, 7] of the contrast material has been suggested as a risk factor for CI-AKI. The first generation of RCM were cheaper, ionic, and hyperosmotic (HOCM > 1 000 mOsm/L) (Table 1). Second-generation RCM are more expensive [18, 38], nonionic, low-osmolality RCM (LOCM: 600–900 mOsm/L) where iodine is covalently bound to an organic component (Table 1). The newest generation of RCM, introduced in the 80s, is isoosmotic (IOCM: 280–320 mOsm/L) with a dimeric structure. Although the osmolality of the RCM was held responsible for CI-AKI previously, the withdrawal of HOCM did not reduce the incidence of CI-AKI [39]. Moreover, clinical studies including a recent meta-analysis [40] did not find differences in the incidence of CI-AKI or renal safety profile between LOCM and HOCM [41, 42]. The* osmolality* of tested RCM did not influence the extent of tubular cell vacuolization in rats [43]. Similarly, our mouse (NMRI) model did not confirm the hypothesis that higher osmolality is more deleterious. Functional (survival, plasma urea) and morphological (tubular damage index) end-points were similar after LOCM (iomeprol) and HOCM (diatrizoate) (unpublished). A recent study comparing HOCM and IOCM concluded that IOCM was retained longer in the kidneys. The explanation for longer renal handling was the higher* viscosity* of IOCM due to their dimer structure and the lack of osmotic diuresis [44]. Resistance to flow is linearly proportional with the viscosity of the fluid and the length of the vessel and inversely proportional to the fourth power of the vessel radius (Poiseuille's law). Thus, viscosity and not osmolality determines flow especially in narrow and long peritubular capillaries [45–47]. In order to reduce toxicity, high viscosity RCM is warmed up to 37°C before administration reducing viscosity with about 50% (Table 1). Despite warming, several studies suggested that dimer IOCM is more toxic than monomer LOCM (Table 1) [45–47]. Furthermore, viscosity increases exponentially in the tubules during the concentrating procedure leading to slower tubular flow [46]. Thus, hydration may be beneficial by reducing urine concentration and viscosity in tubules [44–46]. Interestingly, the comparison of the highest viscosity iohexol (Omnipaque)® and the lowest viscosity metrizoate (isopaque) did not support the role of high viscosity in CI-AKI, as metrizoate caused more renal damage compared to iohexol [48–50]. A recent meta-analysis concluded that there is no difference in nephrotoxicity among various RCM owing to differences in ionicity, osmolality, or viscosity [51]. High viscosity could be a risk factor in insufficiently hydrated patients as hydration may be especially effective in preventing CI-AKI caused by high viscosity IOCM [45].

## 3. Differences between Human and Rodent Kidneys

**Figure 2:**
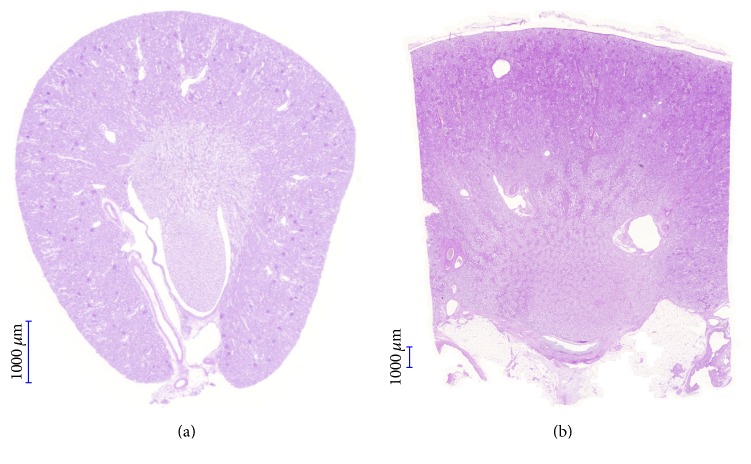
Comparison of mouse and human kidney. The one-papilla mouse kidney has a well developed outer stripe (a) (own picture), whereas this zone is much less prominent in the multipapilla human kidney (b) (courtesy of Attila Fintha, Semmelweis University, 2nd Department of Pathology) (magnification: 10x, PAS staining).

Anatomically, rodents generally have a one-papilla kidney compared to the multi-papilla (4–18) human kidneys. The anatomic zones are similar [52, 53]. Mice have on average 14 000 and rats 22 000–25 000 [54] nephrons whereas humans can vary from 200 000 to 1.8 [55] or up to 2.7 [53] million nephrons. The mouse glomerulus is around 70 *μ*m, whereas the human glomerulus is 200 *μ*m in diameter [52]. The distal tubule has a brush border in mice but not in humans [52]. The outer stripe of the outer medulla in rodents is much more developed than in the human kidney (Figure 2). The supporting connective tissue is more prominent in humans than in mice [52]. Importantly, cortical proximal tubular epithelial cells can be vacuolated in male mice [52]. Furthermore, nephrogenesis is complete in humans at term birth whereas, in rodents, the majority of nephrons are formed after birth [53].

Functionally, the renal concentrating ability is higher in rodents than in humans, especially in mice (4000 mOsm/kg) [56] but not in rabbits (Table 2). The higher concentrating ability in mice is due to the complex, large bundles of vasa recta, which envelop the thin loop of Henle in mice. In humans, loops of Henle and vasa recta are simple and separated. Furthermore, the higher ratio (3 : 1 versus 7 : 1 in human) of long-loop-of-Henle nephrons over short ones contributes to the higher concentrating ability in mice [53]. Also, proteinuria is <150 mg/day and is mainly uromodulin (Tamm-Horsfall protein) in healthy humans, whereas both mouse (0.6–3.1 mg/day) [57] and rat (2–15 mg/day) males (but not females) have considerable proteinuria [58] with an age dependent increase in glomerular filtration of large proteins, such as albumin, and decrease in sex dependent proteins [59]. Age dependent proteinuria is due to age related chronic progressive nephropathy (CPN) in rats [60].

## 4. Rodent Models of CI-AKI

A single injection of iodine containing RCM (5 g/kg ioxilan) induces CI-AKI only in rabbits [87, 88] but does not cause overt kidney damage in rats and mice [74, 89]. Therefore, rabbits offer more reliable model; however, rabbit facilities are not as common as mouse or rat facilities as rabbits are much more expensive to keep. Although tubular epithelial cell vacuolization was described in rats following a single injection of RCM without additional kidney pathology [72, 90, 91], vacuolization alone is not specific enough as a sole end-point to demonstrate CI-AKI. Although functional and histological damage was absent, molecular changes (increased adenosine receptor expressions) were induced by a single iodixanol injection even in mice [92]. To induce a clinically relevant and functionally obvious CI-AKI in rats and mice a combination of injuries is required (Table 3).

The classic rat model of CI-AKI includes inhibition of vasodilators with nitric oxide synthase (NOS) inhibition by 10 mg/kg N*ω*-nitro-L-arginine methyl ester (L-NAME) combined with prostaglandin synthesis (cyclooxygenase (COX) enzyme) inhibition by 10 mg/kg indomethacin [67]. The NO + prostaglandin inhibition rat model was reproduced in mice by Lee et al. [70]. In C57BL/6*J* [personal communication] mice, treatment with 10 mg/kg L-NAME + 10 mg/kg indomethacin + 1 g/kg iohexol (Omnipaque/viscosity: 11.2, osmolality: 780, Table 1) induced a tripling of plasma creatinine [70]. However, a more recent study by Linkermann et al. reported that in C57BL/6*N* mice (Charles River, Germany) unilateral nephrectomy + indomethacin (100 *μ*g/kg) + L-NAME (100 *μ*g/kg) + water deprivation (16 h) + iomeprol (Imeron/viscosity: 8.1, osmolality: 726, Table 1) (250 *μ*L) did not induce any creatinine or urea elevation [81]. For a CI-AKI model the additional insults should not cause major renal injury. Higher doses (>100 *μ*g/kg) of indomethacin with L-NAME induced severe acute renal failure in Linkermann's studies and thus a CI-AKI model was not used [personal communication]. Finally, Linkermann and colleagues applied acute ischemia induced by 30 minutes of bilateral renal pedicle clamping + RCM 24 h after reperfusion as a CI-AKI mouse model [81]. This model provides a reliable method to induce CI-AKI; however it requests a staff experienced in microsurgical procedures. Mice are resistant to several human renal diseases contrary to rats [93]. The slightly lower viscosity and osmolality of iomeprol used by Linkermann et al. do not explain the observed difference between the 2 mouse studies. However, there are substantial genetic and phenotypic substrain differences [94] between C57BL/6J and N mice [95]. Also in renal disease models C57BL/6J mice were resistant to different kidney injuries, such as doxorubicin tubulopathy [96], obesity [97, 98], diet [99], or streptozotocin induced diabetic nephropathy [100] and hepatic, renal, and cardiac fibrosis [101]. In the background genetic differences have been demonstrated [94, 102, 103]. NIH-Swiss [104] or 129/SV [105] mice are even more resistant to renal ischemia-reperfusion injury than C57Bl/6 mice. According to our comparison of J and N C57BL/6 mice, 24 hours after 30 min renal ischemia blood urea was 105 ± 20 mg/dL in J versus 150 ± 24 mg/dL in N mice. Thus, J mice may be less sensitive to renal insults, including hypoxic injury compared to N mice.

Taken together, it is easier to induce CI-AKI in rabbits (single injection of RCM without any additional injury) than in rats and the most severe additional injury is required in mice (Table 3). As the site of urine concentration is the medulla, which is also the site of hypoxic injury in CI-AKI, we hypothesize that high concentrating ability (Table 2) may protect the renal medulla from RCM-induced hypoxic damage in mice. A hypothetic mechanism of protection may be preconditioning to hypoxia by the energy demanding process of establishing the high osmotic gradient between tubular epithelial cells and the medullary interstitium.

## 5. Characteristic Histopathological Changes in CI-AKI

### 5.1. Tubular Vacuolization Is a Histological Marker of CI-AKI

Iodinated contrast media are eliminated almost entirely by glomerular filtration [106]. Filtered RCM becomes concentrated in renal tubules during the concentration process of the primary urine. Thus, tubular epithelial cells are exposed to an increasing concentration of RCM. Consequently, tubular epithelial cell damage should be a leading histopathological event in CI-AKI [25].

**Figure 3:**
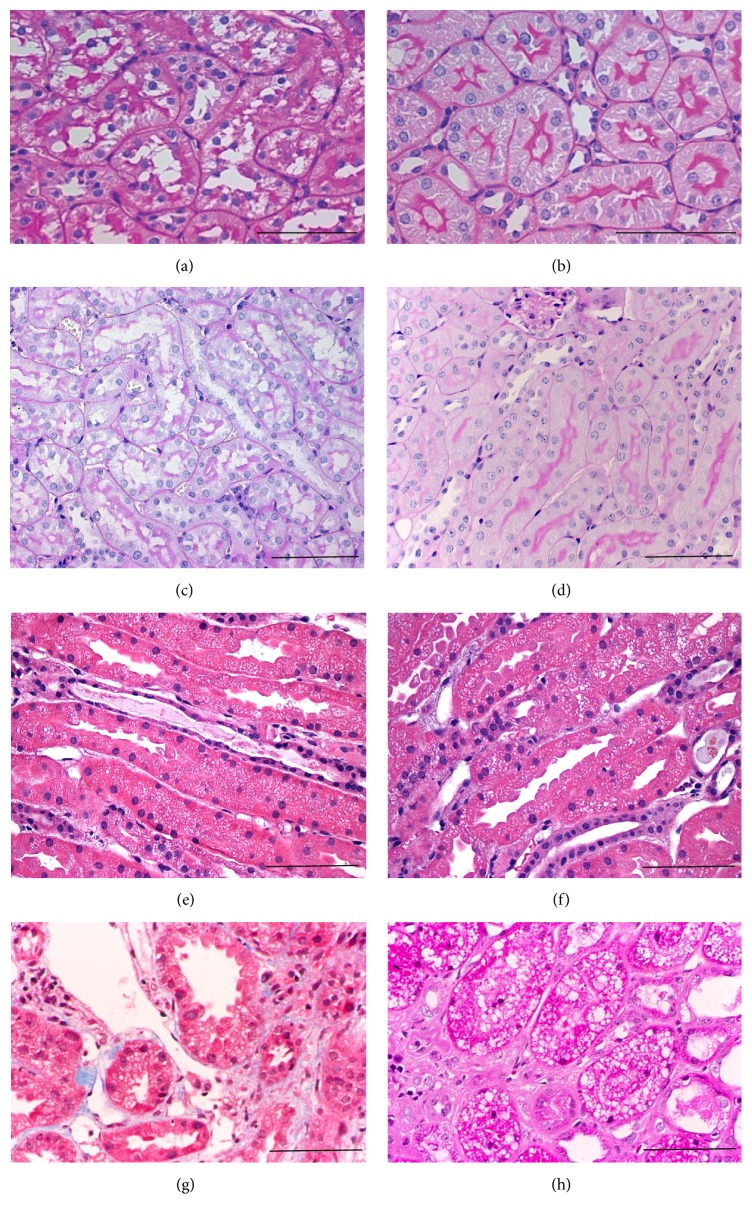
Vacuolization in different rodent models of CI-AKI. (a, b) Tubular cell vacuolization in a CI-AKI rat (Sprague-Dawley) model. (a) Indomethacin + L-NAME + ioversol. (b) Normal rat kidney cortex (PAS, 400x, [31]). (c, d) Hydropic degeneration in mice. (c) Sucrose-induced hydropic degeneration of tubular cells in mouse (NMRI mouse, three ip. injections of 5% sucrose). (d) Normal histology of an intact mouse (PAS, 400x, [32]). (e–h) Vacuolization induced by calcineurin toxicity versus ischemia. (e–g) Isometric vacuolization in calcineurin inhibitor (CNI) toxicity (courtesy of Professor Michael A. Nalesnik, MD, University of Pittsburg, Division of Transplantation Pathology). (h) Coarse, irregular vacuolization following ischemia (HE, 400x, (h) from [33], with permission). Scale bar represents 100 *μ*m.

A general histopathological feature of CI-AKI is* vacuolization of tubular epithelial cells* [42] (Figures 3(a) and 3(b)). Tubular vacuolization is commonly interpreted as a sign of drug toxicity [107]. A single injection of RCM to intact rats induced tubular vacuolization in the absence of other kidney pathologies [72, 90, 91]. Ultrastructural studies of these kidneys suggested that the vacuoles were membrane-bound lysosomes [42, 65, 90]. Although in one study vacuoles were absent if the CT contrast gadolinium DTPA was used, a more recent study did not confirm the absence of vacuoles by CT contrast materials [106]. These reversible, lysosomal alterations primarily detected in the proximal tubules are the* earliest* signs of RCM toxicity [72].

### 5.2. Tubular Vacuolization Does Not Correlate with Renal Function

Vacuolization is often* reversible* even after extremely high dosages of RCM in rats [86, 106] and functional deterioration is absent or mild. In humans, both anuria without vacuolization and diffuse vacuolization without loss of renal functional have been described [107, 108]. Furthermore, vacuolization was absent in rats despite 24 h water deprivation + nephron reduction + high doses of different RCM [107, 108] but was present in another study on rats deprived of water 24 h before RCM injection. Both studies used Wistar rats. The vacuolization almost disappeared 48 hours after application of iobitridol but not after iohexol [73].

*Vacuolization does not correlate with renal function* impairment because

- tubular vacuolization per see does not cause loss of renal function,
- tubular vacuolization resolves spontaneously,
- more severe tubular damage may lead to the shedding of vacuolated cells into the urinary space. New cells replace the shed epithelial cells.

This* discrepancy* between functional and morphological deterioration poses a problem for the histopathological evaluation. Optimally, CI-AKI histopathology should be evaluated within 24–72 hours after induction and a serial evaluation is better than choosing only one time-point.

#### 5.2.1. Possible Other Causes of Tubular Vacuolization

*(1) Physiological Finding or Artifact*. Vacuolization can be a* physiological* finding. In the human kidney, 70% of the juxtaglomerular cells contain vacuoles mostly in the perinuclear area, commonly seen by light microscopy. In mice, vacuolization is a common background finding in cortical epithelium [109] that can indicate a fixation* artifact* or postmortem changes [110]. Vacuolization is related to autolysis or poor fixation and is often observed in survival studies in animals sacrificed in a moribund state [62].

Vacuolization can be* strain or sex dependent* as well. In 2-3% of CD-1 mice lysosomal vacuoles were demonstrated [111]. Vacuoles were present only in male but not female Sprague-Dawley (SD) and Wistar-Han (WH) rats [62].

*(2) Hydropic Vacuolization (“Osmotic Nephrosis”)*. Tubular vacuolization or hydropic degeneration [112] is a histological sign of the so-called “osmotic nephrosis.” The name “osmotic nephrosis” comes from the initial description by* Allen* in 1951. He observed large vacuoles in tubular epithelial cells following hypertonic sucrose infusion in rabbits [113] and humans [114, 115] and interpreted the vacuolization as the result of an osmotic gradient between the tubular lumen and tubular cells [116]. Hydropic vacuolization develops after intravenous injection of substances eliminated by the kidney, such as RCM [72, 114], polyethylene-glycol- (PEG-) conjugated proteins [117], hydroxy-ethyl-starch (HES) [118–120], dextran [121, 122], sucrose [43], mannitol [123], glucose [124], glycerol [125], sorbitol [126], inulin [127], or sugar (sucrose [128–130] or maltose [131]) stabilized intravenous immunoglobulin (IVIG) solutions. According to our experience (Figures 3(c) and 3(d)) different concentrations and repeated intraperitoneal doses of sucrose or maltose induced tubular vacuolization dose dependently. Sucrose-stabilized IVIG had a similar effect [32].

Although the condition was named after the swelling of tubular epithelial cells, the reason for this swelling is not osmotic pressure but the formation of vacuoles [114]. Ultrastructural studies demonstrated that the vacuoles are lysosomes. The agents causing hydropic vacuolization are taken up by tubular epithelial cells through* pinocytosis* [107, 108] already 5 min after injection and appear as small vesicles on electron microscopy [72]. Pinocytotic vesicles fuse together and fuse with lysosomes forming the larger vacuoles, detectable by light microscopy [114, 132]. Thus, there is consensus about the rejection of the osmotic hypothesis [106–108], but the misleading term [107] is still in use [114].

Hydropic vacuolization is reversible [43] and renal function loss is often missing [32, 43, 117]. Initially fine apical vesicles become large by fusion and dislocate the nucleus at advanced stages, which can be accompanied by functional deterioration. N-Acetyl-*β*-D-glucosaminidase (NAG) and lactate dehydrogenase (LDH) are damage markers of proximal tubular epithelial cells [133–135]. RCM injection induced a dose-dependent vacuolization with an increase in urinary NAG and LDH excretion that correlated with vacuolization suggesting a pathophysiological role for vacuoles in CI-AKI [106].

Osmotic diuresis (e.g., induced by* mannitol*) has been even suggested as a protective mechanism against CI-AKI by accelerating elimination of the contrast material from the tubular lumen [136]. Our experience in mice did not support any beneficial effects of mannitol. In a mouse model, 22-minute renal ischemia + Omnipaque (8 mL/kg) was followed by a 3.2% mannitol infusion at 12 mL/kg per hour but did not provide any functional (urea retention, NGAL excretion) or morphological protection against CI-AKI (unpublished). Similarly, a recent meta-analysis concluded that intravenous mannitol did not have additional benefits over hydration in AKI patients and mannitol was even detrimental in CI-AKI patients [137].

*(3) Further Causes of Tubular Vacuolization*. A special form of tubular epithelial cell vacuolization is phospholipidosis* (PLD)*: a reversible accumulation of polar phospholipids in different organs such as the kidney, liver, lung, brain, and lymphoid tissues [91]. PLD can be caused by certain drugs (such as antibiotics (e.g., aminoglycosides [138]) or tricyclic antidepressants). These cationic drugs accumulate in lysosomes [72, 91]. The morphological hallmark in PLD is the lamellar structure of the lysosomes* (lamellar bodies)*. Functional deterioration is usually absent in PLD [125] as it is in other forms of hydropic vacuolization.

Calcineurin inhibitors* (CNI)* such as cyclosporine A (CsA) or tacrolimus (Tac) also cause vacuolization of tubular cells [107], which appear similar to the previous pathologies, although more* isometric* (Figures 3(e)–3(h)) [112, 114]. However, isometric vacuoles were described after RCM or mannitol administration as well [107, 139]. The vacuoles in CNI toxicity are not lysosomes but are dilated endoplasmic reticulum due to immune mediated tubular injury [107] as verified by electron microscopy [114, 140]. In contrast to the causes of tubular vacuolization described above, CNI toxicity is accompanied by* loss of renal function* [141] due to renal vascular injury and/or thrombotic microangiopathy.

#### 5.2.2. Pathomechanism of Contrast-Induced Tubular Vacuolization

CI-AKI vacuoles were located primarily in the proximal tubules and are lysosomes [44, 142]. Iodine was retained in the renal cortex [44] and the RCM was abundant in vacuoles 7 days [143, 144] and was still present 28 days [143] after administration, besides normal renal function [44, 144]. Thus, CI-AKI vacuoles are a consequence of RCM reabsorption.

Despite high RCM doses, vacuoles were absent in healthy kidneys and no tubular necrosis or atrophy developed unless there was some concomitant or predisposing renal damage [108]. RCM induced AKI in transplanted kidneys during an acute rejection episode but not during a rejection-free period [108]. Functional impairment can be absent as long as proximal tubular vacuolization may be within the kidney's functional reserve capacity [144]. The duration of vacuoles' presence depends on the digestibility of the pinocytosed substance [114, 132]. Preexisting (e.g., hypoxic or diabetic) kidney damage can substantially delay lysosomal digestion [114] and, thus, prolong the presence of the vacuoles.

*In summary*, tubular vacuolization is a consequence of pinocytosis of the RCM and lysosomal fusion. Tubular vacuolization is an early sign of CI-AKI but without comorbidities it does not progress to tubular cell necrosis [114]. Although tubular vacuolization is a direct consequence of the RCM present in the tubular lumen, it is not specific—as several other pathologies can cause it and it is not sensitive—as there is little correlation with renal function [18]. Taken together, vacuolization is the earliest marker of CI-AKI. However, its lack of specificity, the lack of correlation with renal function, and its tendency to disappear prevent the use of tubular vacuolization as a sole hallmark of CI-AKI.

## 6. Further Pathological Markers of CI-AKI

### 6.1. Hypoxic Damage

It is generally accepted that hypoxia plays an important role in the development of CI-AKI [124]. In healthy rabbits, a single injection of RCM induced medullary hypoxia due to reduction of renal blood flow (RBF) as demonstrated by magnetic resonance studies [145–148]. Already in the 70s it has been described that RCM injection was associated with a 30–50% decline of para-aminohippurate (PAH) extraction in dogs [149, 150] and humans [151] suggesting a reduction in RBF [18, 149]. Measurements with electromagnetic flow meters allowed a more detailed analysis and demonstrated an initial transient (<30 sec) vasodilation before the prolonged (5–15 min) vasoconstriction [152] with substantial decrease in RBF and glomerular filtration (GFR). Vasoconstriction of peritubular capillaries causes prolonged medullary hypoxia [21, 146–148, 152]. However, the mechanism of vasoconstriction is not clear. Previous theories about osmotic injury or high viscosity are not supported by recent studies. Similarly, a central role of the renin-angiotensin-aldosterone system (RAAS) is not supported by the facts that the decrease in RBF preceded the increase in plasma renin activity (PRA) and angiotensin-II antagonists did not inhibit the RCM-induced vasoconstriction [46, 152]. On the other hand, nitric oxide and prostanoids protect from RCM-induced vasoconstriction [67]. Inhibition of these systems is often used in CI-AKI models [65, 66, 68, 69]. Thus, reduction of dilator prostanoids and the NO system may be involved in RCM-induced medullary hypoxia.

#### 6.1.1. Endothelial Damage

The contribution of reactive oxygen species (ROS) to CI-AKI pathology is widely accepted. ROS contribute to intrarenal vasoconstriction by scavenging NO. Endothelin also contributes to the vasoconstriction [20]. Vascular endothelial injury has been suggested in the background of contrast-induced vasoconstriction [25]. The endothelial cells are the first to come in contact with intravenously injected RCM [25]. Direct endothelial cell damage was observed by electron microscopy in rat aortic endothelial cells [153]. Endothelial damage in peritubular capillaries by RCM directly or through ROS can be an important driving force of the medullary hypoxia.

### 6.2. Patchy Nature of Hypoxic Damage

Tubular epithelial cells are the most sensitive to hypoxia. However, there are substantial regional differences in the severity of hypoxia. As detailed below, there is an inverse relationship between oxygen supply and need from outer cortex to inner medulla. Furthermore, with increasing distance from vasa recta oxygenation is decreasing. Due to these regional differences of oxygen supply and demand, histological changes are often focal or patchy and inhomogeneous in the postischemic or CI-AKI kidney. This* inhomogeneity* may explain negative biopsy results despite severe functional deterioration in humans and point to the necessity of systemic evaluation of whole kidney cross sections in rodent experiments.

#### 6.2.1. Tubular Hypoxia

**Figure 4:**
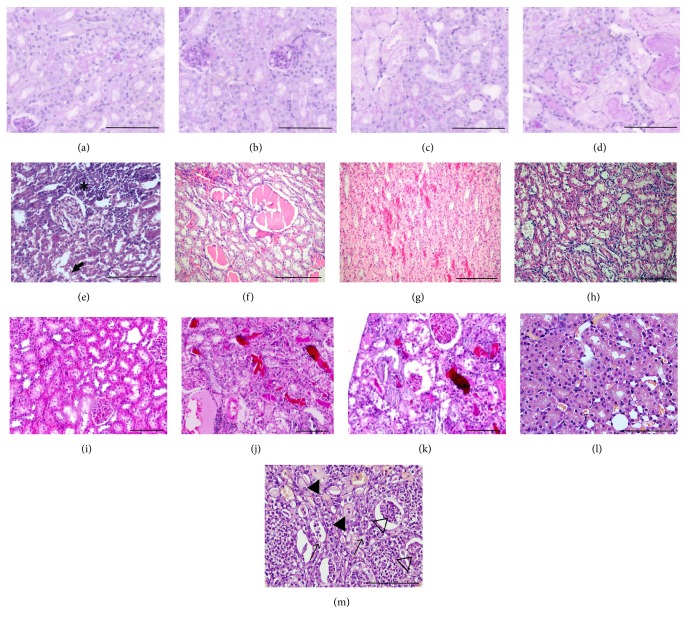
Histopathology of CI-AKI models with RCM administration and hypoxia. (a–d) RCM + renal ischemia mouse model (unpublished own data) (PAS, 400x). (a, b) Hypoxic tubular damage in mice 24 h after Omnipaque 350 iv. alone but no additional ischemia: besides vacuolization and mild tubular cell injury, no necrosis can be observed. Proximal tubuli have an intact brush border. (c, d) Ischemic changes in mice after 22 min ischemia + Omnipaque 350 iv. + 24 h reperfusion: more severe tubular damage, flattening of tubular epithelial cells, loss of nuclei, dilation of tubular lumen, and cast formation demonstrate tubular necrosis. (e) RCM + indomethacin rat model. Necrotic tubular cells (arrow) and inflammatory cell infiltration (*∗*). (RCM (10 mL/kg iomeprol), indomethacin (10 mg/kg), and HE, 200x, [34]) (f–h) RCM + dehydration rat model. Tubular casts (f, g), medullary congestion (g), and tubular necrosis (h) (72 h dehydration + RCM, HE, 200x, [35] with permission). (i–k) Glycerol based CI-AKI model. (i) Normal rat kidney. (j) Glycerol (im) + 24 h water deprivation. (k) Glycerol + RCM. Tubular necrosis and cast formation are more sever after combined injury (HE, 200x, from [36] with permission). (l, m) Combined model of diabetic nephropathy + RCM. (l) Tubular vacuolar degeneration (arrows), necrosis, hyaline casts (filled triangles), and cellular casts (hollow triangles) and inflammatory infiltration in contrast-treated diabetic and (m) normal rat kidney medulla (HE, 200x). From [37] with permission. Scale bar represents 100 *μ*m.

Renal tubular epithelial cells are the most sensitive to hypoxia due to their high metabolic demand. Furthermore, due to the countercurrent circulatory system of the kidney, the oxygen supply decreases towards the medulla as the oxygen demand increases. Thus, tubular epithelial cells are the first to suffer from hypoxic damage. Despite many papers describing hypoxia as an important contributor to CI-AKI,* tubular cell necrosis* is usually* absent*, despite the presence of proximal tubule vacuolization [72, 106, 154, 155]. RCM per see do not cause necrosis (Figures 4(a) and 4(b)). Necrosis was present only if RCM was combined with other hypoxia triggers (Figures 4(c) and 4(d)). Thus, direct toxic injury of RCM to TEC is not likely in healthy kidneys; however, the primary targets of renal hypoxia are TEC; thus a hypoxic injury may sensitize TEC to RCM toxicity.

### 6.3. Tubular Toxicity

Direct tubular toxicity of RCM is considered to participate in the pathomechanism of CI-AKI [5, 14, 156]. However, most of the direct toxicity data are based on* in vitro* studies, reporting about reduced cell viability in animal [157–162] or human cell cultures [33, 163]. In suspended rabbit tubular epithelial cells [164] or isolated proximal tubule segments [165] RCM toxicity was observed only with concomitant ischemia [18]. Furthermore, in different tubular cell cultures RCM induced mitochondrial swelling [65] and DNA fragmentation and/or apoptosis [46, 163, 166–169]. Also, in human CI-AKI patients, tubular cells were found in the urine [170]. However, incubation with RCM at therapeutic concentrations did not induce cell death, despite rapid uptake of RCM in cultured primary or immortalized tubular epithelial cells or isolated mouse tubules [81]. Therefore, the suggested mechanism of RCM-induced cytotoxicity* in vivo* [170] is apoptosis induced by oxidative damage to the tubular epithelial cell membrane by reactive oxygen species (ROS) [20].

## 7. Further Histological Changes Related Primarily to the Model and Not to RCM Injection

### 7.1. Ischemia-Reperfusion Injury Induced by Renal Clamping

Rodent CI-AKI models apply renal hypoxia to aggravate the kidney damage that is subclinical if RCM is given alone (Table 3). Although RCM alone does not cause necrosis, the addition of hypoxia culminates in acute tubular necrosis (ATN) (Figures 4(c) and 4(d)) [171].

In these models a control group with renal ischemia/hypoxia but without RCM is necessary to differentiate the effects of RCM from clamping. The severity of ischemia/hypoxia has to be adjusted as too severe damage may prohibit the evaluation of RCM-induced pathology, whereas if the model is too mild, kidneys may remain unaffected.

A disadvantage is the fundamental difference between rodent renal ischemia-reperfusion injury and human hypoxic AKI. Important differences include the following: complete cessation of blood flow (anoxia) in rodent models versus reduced blood flow (hypoxia) in humans, and temperature during the anoxia/hypoxia is close to physiologic in rodent models, whereas it is often reduced in human AKI. Warm ischemia primarily affects the cortex and the outer stripe, whereas cold ischemia damages the inner stripe and the renal papilla [171]. Our own observations confirm that in the mouse warm ischemia-reperfusion model cortex and outer stripe of the medulla are the primary localization of tubular injury [172] as opposed to papillary necrosis in several forms of human AKI (CI-AKI, non-steroid induced (analgesic) nephropathy or AKI accompanying prolonged surgery). The most affected outer stripe of the outer medulla in rodent models is much less developed in the human kidney [171].

In healthy (sham operated) kidneys tubules have narrow lumen in the cortex (Figure 4(a)) and intact brush border in the outer stripe (Figure 4(b)). Following 30 minutes of ischemia and 24 h reperfusion, tubuli in the cortex are dilated (Figure 4(c)) and filled with PAS positive hyaline in the outer stripe (Figure 4(g)) with loss of nuclei and cellular structure. On the other hand, cells of the inner stripe do not show morphologic damage (Figure 4(k)).

### 7.2. Ischemic-Injury Aggravated by Prostaglandin Inhibition (Indomethacin)

A common pathomechanism in the nephrotoxicity of nonsteroidal anti-inflammatory drugs (*NSAIDs*, e.g., indomethacin, analgesic nephropathy), calcineurin inhibitor (CNI) immunosuppressives (CSA, Tac) (CNI nephropathy), and iodinated RCM (CI-AKI) is medullary hypoxia [173]. Dilator prostanoids serve as the last reserve for renal vasodilation [174] in injured kidneys such as in diabetic nephropathy or in a dehydrated state [82]. The suppression of prostanoids amplifies the medullary hypoxia both in CI-AKI and in analgesic nephropathy. In murine models of CI-AKI, with RCM and indomethacin, proximal tubular vacuolization is accompanied by medullary tubular necrosis and cast formation [20, 65] (Figure 4(e)). Thus, the prostaglandin (and NOS) inhibition models have the advantage over renal clamping that they resemble more human CI-AKI pathology as the injury is located predominant to the renal medulla [171].

### 7.3. Dehydration

Prolonged (72 h) dehydration combined with RCM causes CI-AKI in mice [75] and rats [35]. Dehydration alone significantly reduced renal cortical antioxidant (superoxide-dismutase [SOD] and catalase [CAT]) expression in rats [74]. The leading histopathological changes were tubular necrosis with cast formation and medullary vascular congestion (Figures 4(f)–4(h)) [35, 75].

### 7.4. Glycerol Induced Rhabdomyolysis

Intramuscular glycerol injection-induced rhabdomyolysis is a model of acute renal failure. As the hydration status of the body during rhabdomyolysis significantly influences the development of renal failure, 24-hour water deprivation precedes glycerol injection in this model [77]. Histological damage includes tubular necrosis: hyaline and hemorrhagic casts in cortex and medulla aggravated by the addition of RCM (Figures 4(i)–4(k)) [78].

### 7.5. Tubulointerstitial Fibrosis

CKD is an important risk factor for CI-AKI. Thus, CKD rodent models plus iv. RCM injection is also used to model CI-AKI [82, 84, 85, 175]. For example, RCM induces tubular necrosis in diabetic nephropathy kidneys (Figures 4(l) and 4(m)) [37]. A yes-or-no phenomenon regarding RCM dose has been reported in the diabetic CI-AKI model as 8 mL/kg or 10 mL/kg iopromide did not but 12 or 16 mL/kg did induce renal functional decline [9, 37].

## 8. Conclusion

In summary, the most specific histopathological lesions in rodent CI-AKI models are vacuolization of tubular epithelial cells and medullary hypoxia. Necrosis is only present if other hypoxia triggers are also applied as part of the model. As histopathologic changes lack specificity it is a relevant marker but not sufficient enough. Thus, further functional parameters and molecular biomarkers should be included in CI-AKI animal studies for a comprehensive analysis of disease progression. As the injection of RCM alone does not cause overt AKI in rodents, multiple insults are necessary for inducing histopathological and functional decline. The difference in sensitivity between species and the correlation with renal concentrating ability suggests that high concentrating ability may protect from CI-AKI.

## Figures and Tables

**Table 1:** Viscosity and osmolality of the 3 generations of radiocontrast materials (RCM). The iodine/molecule ratio is 1.5 : 1 in high-osmolality contrast media (HOCM), 3 : 1 in LOCM (tri-iodinated molecules), and 6 : 1 in IOCM dimers [61].

Osmol. group	Name		Chem struct	Viscosity (mPa)		Osmolality (m)	Year intro.
(intro.)	Chemical	Brand		(20°C)	(37°C)	mOsm/kg H_2_O	
Isoosmotic (IOCM) (1990s)	Iodixanol	Visipaque	Nonionic dimer	26.6	11.1	290	1996
	Iotrolan	Iovist		6.8	9.5	320	1989
Low (LOCM) (1980s)	Ioxaglate	Hexabrix	Ionic dimer	15.7	7.5	600	1985
	Ioxilan	Oxilan	Nonionic monomer	16.3	7.8	695	1995
	Iomeprol	Imeron		15.6	8.1	726	1994
	Iopromide	Ultravist		22	9.5	770	1995
	Iohexol	Omnipaque		20.4	11.2	780	1985
	Ioversol	Optiray		18	8.5	792	1988
	Iopamidol	Isovue		20.9	9.8	796	1997
	Iobitridol	Xenetix		21	10	915	1994
High (HOCM) (1950s)	Diatrizoate	Crystographin Hypaque	Ionic monomer	18.5	8.4	2000, 1550	1955
	Metrizoate	Isopaque		NA	3.4	2100	1959
	Iothalamate	Conray		NA	9	2400	1962

**Table 2:** Differences between human and rodent kidney, summarized from [53] with additional data for human [52, 53, 62], mouse [42, 56, 63], and rabbit and rat [53, 64] species.

	Human	Rabbit	Rat	Mouse
Number of papillas	7–9	1	1	1
Number of nephrons	0.2–2 million	30 000	25–35 000	10–14 000
Concentrating ability (mOsmol/kg)	1200	1400	3000	4000
Glomerular diameter (*μ*m)	200	140	120	73

**Table 3:** Rat and mouse models of CI-AKI.

Injury type (besides RCM injection)	Species	Advantage	Disadvantage	Ref.
*Inhibition of vasodilators*		Pronounced medullary hypoxia	Multiple insults	
Indomethacin (+salt depletion ± UNX)	Rat	Complex, clin. relevant	CPN for all rat models	[65–69]
Indomethacin + L-NAME	Rat	Medullary hypoxia		
Indomethacin + L-NAME	Mouse	pathomechanistic	High drug dose needed	[70, 71]
*Water deprivation (dehydration)*		Dehydration amplifies injury	Hydration state affects CI-AKI progression	
Dehydration (24 h)	Rat			[72, 73]
Dehydration (72 h)	Mouse, Rat			[35, 74, 75]
Dehydration (24 h) + eNOS deficiency (KO)	Mouse			[76]
Dehydration (24 h) + Indomethacin + furosemide	Rat			[34]
Dehydration (24 h) + glycerol rhabdomyolysis	Rat			[36, 77–80]
*Surgical kidney injury models*		Reliable models	Microsurgery experience	
*Acute kidney injury (AKI)*		Short duration	Species differences	
Ischemia-reperfusion	Mouse			[81]
*Chronic kidney disease (CKD)*		Clinical relevance	Chronic protocol	
Diabetes (streptozotocin: STZ)	Rat			[82, 83]
5/6 nephrectomies + dehydration (48 h)	Rat			[84, 85]
Long term cholesterol feeding	Rat			[86]

clin.: clinically, UNX: Uninephrectomy, CPN: chronic progressive nephropathy, and eNOS: endothelial nitrogen monoxide synthase.
